# Coapting COAPT and MOMENTUM 3: Advancing heart failure, secondary mitral regurgitation, and the crossroads of therapies

**DOI:** 10.1016/j.jhlto.2024.100052

**Published:** 2024-01-07

**Authors:** Pierre-Emmanuel Noly, Jennifer A. Cowger, Thomas M. Cascino

**Affiliations:** a University of Michigan, Ann Arbor, Michigan; b University of Montreal, Montreal, Quebec, Canada; c Henry Ford Hospital, Detroit, Michigan

Driven by the favorable results of the Cardiovascular Outcomes Assessment of the MitraClip Percutaneous Therapy for Heart Failure Patients with Functional Mitral Regurgitation (COAPT) trial,^1^ there has been widespread adoption of transcatheter edge-to-edge repair (TEER) to treat patients with advanced heart failure with reduced ejection fraction (HFrEF) and severe secondary mitral regurgitation. Since the publication of COAPT, the use of TEER has subsequently increased exponentially, including many sites that do not perform advanced therapies.^2^ It is anticipated that the use will continue to grow following the inclusion of TEER in the heart failure guidelines as an adjunct to care after optimizing guideline-directed medical therapy (GDMT) in patients with persistent severe secondary mitral regurgitation.^3^

As the medical and device therapies for HFrEF continue to expand, there is a need to ensure patients are empowered to make fully informed decisions that are aligned with their values and goals.^4^ Central to shared decision-making is a complete comprehension of all potential care options available with the additional knowledge of the anticipated outcomes associated with each intervention (e.g., survival, complications, and impact on quality of life) based on clinical trial and registry data. For the patient with advanced HFrEF and severe secondary mitral regurgitation, there is a potential overlap in candidacy for TEER technologies and durable left ventricular assist device (LVAD) support. In this situation, patient characteristics and goals of care must be used to modify projected therapeutic response beyond clinical trial averages so patients and referring providers are knowledgeable about patient-specific outcome estimates from the application of each therapy.

Following the COAPT trial,^1^ heart failure guidelines recommend the use of TEER in patients with persistent severe secondary mitral regurgitation, NYHA class II to IV symptoms, a left ventricular ejection fraction (LVEF) between 20% and 50%, a left ventricular end-diastolic diameter (LVEDD) ≤70 mm, and a systolic pulmonary arterial pressure of <70 mm Hg despite optimal guideline-directed medical therapies.^3^ In contrast, presently approved durable LVAD therapy is indicated for patients with NYHA class IV symptoms, inotrope dependence, or a cardiac index <2.2 ml/min/m^2^ while not on inotropes, and failure of optimal medical therapy. While the TEER and durable LVAD patient populations may at first seem very different, a review of the baseline characteristics and outcomes from the clinical trials that resulted in the approval of both therapies suggests that there is potential overlap in the therapeutic candidacy, especially at lower LVEF and higher LVEDD values. While TEER has been shown to improve survival compared to medical management alone, as discussed below, it may not be the optimal therapeutic decision for patients with advancing heart failure if the patient’s goals are to live longer, feel better, and achieve more functional improvement.

## Patient Phenotypes in COAPT and MOMENTUM 3

Select baseline characteristics of the treatment groups from the COAPT and MOMENTUM 3 trials are shown in Table 1. In the COAPT trial, the average patient composite profile was that of a 72-year-old with an ischemic cardiomyopathy presenting with persistent NYHA class III symptoms, a hospitalization in the past year, a B-type natriuretic peptide of 1014 pg/ml, creatinine clearance of 51 ml/min, and additional echocardiogram findings including severe mitral regurgitation, an LVEDD of 62 mm, and LVEF of 30%.^1^ Recognizing that LVEF will often decrease with reduction in secondary mitral regurgitation, this patient profile would not just be representative of the average COAPT trial patient, but will also overlap with inclusion criteria in the Multicenter Study of MagLev Technology in Patients Undergoing Mechanical Circulatory Support Therapy with HeartMate 3 (MOMENTUM 3) trial.^5^ If the patient has had a recent hospitalization for heart failure (NYHA IV), the patient would also meet the Centers for Medicare and Medicaid Services national coverage decision criteria for durable LVAD therapy.^6^

**Table 1:** Select Baseline Characteristics of Patients in COAPT and MOMENTUM 3 Trials

Characteristic	COAPT TEER N = 302	MOMENTUM 3 HeartMate 3 N = 516
Age, years	71.7 ± 11.8	59 ± 12
Male sex, N (%)	201 (66.6)	411 (79.7)
Ischemic cardiomyopathy	184 (60.9)	216 (41.9)
Moderate-severe MR, N (%)	302 (100)	217 (42.1)
Left ventricular ejection fraction, %	31.3 ± 9.1	17.3 ± 5.1
Medical therapy at baseline, N (%)		
Beta-blocker	275 (91.1)	284 (55.0)
ACEI, ARB or ARNI	216 (71.5)	158 (30.6)
Mineralocorticoid receptor antagonist	153 (50.7)	NR
Diuretic	270 (89.4)	436 (84.5)

Abbreviations: ACEI, ace inhibitor; ARB, angiotensin receptor blocker; ARNI, angiotensin receptor/neprilysin inhibitor; NR, not reported.

Data are presented as means ± SD or N (%).

## Outcomes in COAPT vs MOMENTUM 3

**Figure 1:**
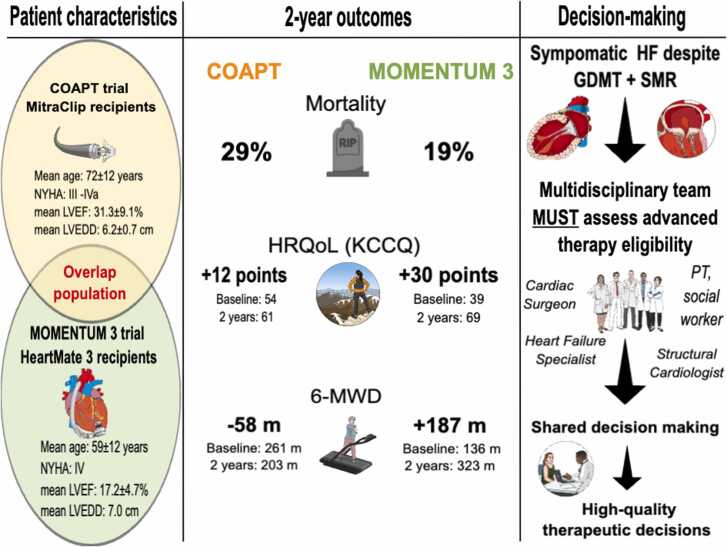
Understanding patients and outcomes after TEER and LVAD and the opportunity to improve shared decision-making. Compared to patients undergoing TEER, similar patients would be expected to have substantial gains in functional status, quality of life, and mortality with LVAD therapy. There is a need to ensure a multidisciplinary team assesses patients prior to LVAD with consideration of advanced therapies to empower patients to make high-quality therapeutic decisions for themselves. 6-MWD, six-minute walk distance; COAPT, Cardiovascular Outcomes Assessment of the MitraClip Percutaneous Therapy for Heart Failure Patients with Functional Mitral Regurgitation; GDMT, guideline-directed medical therapy; HF, heart failure; HRQoL, health-related quality of life; KCCQ, Kansas City Cardiomyopathy Questionaire; LVAD, left ventricular assist device; LVEDD, left ventricular end diastolic diameter; LVEF, left ventricular ejection fraction; m, meter; MOMENTUM 3, Multicenter Study of MagLev Technology in Patients Undergoing Mechanical Circulatory Support Therapy with HeartMate 3; NYHA, New York Heart Association; PT, physical therapy; SMR, secondary mitral regurgitation; TEER, transcatheter edge-to-edge repair.

While patients enrolled in the MOMENTUM 3 trial overall had more advanced stages of heart failure compared to those in the COAPT trial, MOMENTUM 3 patients achieved numerically greater improvements in survival, functional status, and health-related quality of life (HRQoL) when compared with COAPT trial patients (Figure 1). The 2-year mortality in COAPT was 29.1% with TEER and 46% in the medical management arm, compared to 19% in the HeartMate 3 group in the MOMENTUM 3 trial. In addition, patients in MOMENTUM 3 achieved both greater relative and absolute gains in the Kansas City Cardiomyopathy Questionnaire as well as improved overall 6-minute walk distances from 6 months to 2 years.^1^, ^5^ At 5 years, the mortality in COAPT was 57.3% with TEER^7^ compared to 41.6% in the HeartMate 3 cohort^8^ despite a higher risk preoperative phenotype. Thus, it is reasonable to hypothesize that durable LVAD therapy likely offers superior survival compared to TEER in patients with advanced HFrEF and may offer gains in patient-reported outcomes and functional capacity. These gains must be considered at the patient level, using the patient’s best estimate of the quality of life with durable LVAD vs TEER and readmission risks for LVAD-related complications (e.g., bleeding and infection) vs progression of heart failure symptomatology with TEER.

## Knowledge gaps in the field

Despite initially presumably targeting different patient populations, both TEER and durable LVAD have similarly had therapeutic expansion outside national coverage decisions. For example, LVAD is applied to patients with NYHA III symptoms without inotrope dependence,^9^ and TEER has been utilized in patients with an LVEF <20% and cardiogenic shock.^10^ The latter is particularly concerning given that unlike COAPT, the Percutaneous Repair with the MitraClip Device for Severe Functional/Secondary Mitral Regurgitation trial found no benefit of TEER for persistent mitral regurgitation, possibly secondary to higher-risk patient phenotypes with features of advanced ventricular remodeling (e.g., larger LVEDD).^11^ Knowing this, there is a necessity to ensure that indication creep does not occur for TEER therapies, particularly among patients with more advanced heart failure (e.g., dilated LVs and severe LVEF reductions or signs of cardiogenic shock) for whom there is no evidence of benefit from TEER, and there is clear evidence of benefit from advanced therapies including durable LVAD. The use of TEER in patients eligible for LVAD is particularly concerning as limited data suggest that outcomes are poor when LVAD is done after TEER, possibly because of a delay in initiating the appropriate therapy.^12^ While a randomized clinical trial of TEER vs LVAD in patients with advanced HFrEF is unlikely, registry monitoring of the real-world application of TEER outside of clinical trials may eventually allow a more rigorous comparison of TEER vs LVAD outcomes and provide a better understanding of the impact of the TEER as a bridge to durable LVAD or transplant to identify which subpopulation of advancing heart failure patients are most likely to gain benefit from one intervention vs the other (e.g., a patient with a larger regurgitant volume may be more likely expected to derive benefit for a given LVEF). Adequate and nonbiased capture of morbidity, mortality, and patient-reported outcomes at successive time points will be necessary to compare TEER and durable LVAD interventions.

## Optimizing shared decision making

While the less invasive nature of TEER may appeal to patients if not presented with the full spectrum of options and outcomes, we believe that a multidisciplinary heart team, including an advanced heart failure specialist, should evaluate patients with HFrEF and persistent symptoms after optimization of GDMT to discuss next steps. In addition to confirming that a patient has received optimal GDMT, the advanced heart failure physician has comprehensive knowledge about durable LVAD, transplant, and TEER, including features that would support worse outcome after TEER (e.g., LVEF of less <30% with a dilated LVEDD, pulmonary hypertension, or end-organ dysfunction) that is necessary to decide on appropriate therapies. This discussion and communication with patients should acknowledge the uncertainty about receiving an LVAD after TEER and the comparatively improved survival, functional status, and HRQoL that occurs after advanced therapies, including LVAD. Communication of the comprehensive evaluation to the patient is necessary to enable high-quality therapeutic decisions. Facilitating care through shared decision-making discussions will ensure the momentum of the COAPT trial does not negatively impact our patients' opportunities to live better and longer with advancing HFrEF.

## Disclosure statement

The authors declare the following financial interests/personal relationships which may be considered as potential competing interests: Thomas Cascino reports a relationship with National Heart Lung and Blood Institute that includes funding grants. Dr Cascino reported receiving grants from Johnson & Johnson outside the submitted work. Dr Cowger reported receiving consultant fees from the Abbott Publication Committee during the conduct of the study; fees from Abbott Inc for membership on the advisory board, travel-related compensation, and as a speaker (all for LVAD devices); serving on the steering committee for Tendyne and Cephea Valves, and principal investigator and consultant fees from Medtronic (HVAD device); receiving nonfinancial support from BiVACOR data safety and monitoring board; receiving nonfinancial support as a consultant from Corwave outside the submitted work; and Henry Ford Health received money in support of the MagLev Technology in Patients Undergoing Mechanical Circulatory Support Therapy With HeartMate 3 clinical trial and the ARIES trial, both of which are studies of the HeartMate 3 LVAD.

Authors would like to thank Drs Francis D. Pagani and Donald S. Likosky for their important contributions to the paper.

The project was supported by funding from the 10.13039/100000002National Institutes of Health, 10.13039/100000050National Heart, Lung, and Blood Institute K12 HL138039-02 to Dr Cascino.
